# Evaluating predictors of mortality in octogenarians undergoing urgent or emergent trauma laparotomy

**DOI:** 10.1007/s00068-024-02635-3

**Published:** 2024-10-16

**Authors:** Jordan G. Shin, Jeffry Nahmias, Elliot Silver, Robert Painter, Shaina Sedighim, Flora Park, Areg Grigorian

**Affiliations:** 1 Department of Surgery, Division of Trauma, Burns and Surgical Critical Care, University of California, Irvine, Orange, CA USA; 2 Division of Trauma, Burns and Surgical Critical Care, Department of Surgery, University of California, Irvine Medical Center, 333 The City Blvd West, Suite 1600, Orange, CA USA

**Keywords:** Octogenarian trauma patients, Octogenarian operative management, Urgent laparotomy, Emergent laparotomy

## Abstract

**Purpose:**

This study aimed to identify associated risk factors for mortality in octogenarian trauma patients undergoing urgent or emergent laparotomy (UEL).

**Methods:**

Trauma patients ages 80–89 years-old undergoing UEL within 6-hours of arrival were included. A multivariable logistic regression analysis was performed to determine associated risk of mortality.

**Results:**

From 701 octogenarians undergoing UEL, 324 (46.2%) died. Compared to survivors, UEL octogenarians who died had higher rates of cirrhosis (3.5% vs. 1.1%, *p* = 0.028), injuries to the brain (17.3% vs. 5.6%, *p* < 0.001), heart (8.6% vs. 1.6%, *p* < 0.001), and lung (57.4% vs. 23.9%, *p* < 0.001) and lower rates of functional independence (6.4% vs. 12.6%, *p* = 0.007). The strongest independent associated patient-related risk factor for death was cirrhosis (OR 8.28, CI 2.25–30.46, *p* = 0.001). However, undergoing concurrent thoracotomy increased risk of death significantly (OR 16.59, CI 2.07-132.76, *p* = 0.008). Functional independence was not associated with mortality (*p* > 0.05).

**Conclusion:**

This national analysis emphasizes the need to identify and manage pre-existing conditions like cirrhosis and not determine futility based on pre-trauma functional status alone. Concurrent thoracotomy for hemorrhage control increases risk of death over 16-fold.

## Key summary points

**Aim:**

What are the key risk factors of mortality in octogenarian patients undergoing an urgent or emergent laparotomy?

**Findings:**

Pre-existing cirrhosis was the strongest associated risk factor of mortality in this trauma age group. Functional status was not identified as an independent associated risk factor of mortality.

**Message:**

As the global population ages with an increase in older trauma patients requiring emergency surgeries, it is important to identify surgical outcomes and risk factors for poor outcomes in this demographic.

## Introduction

The global demographic landscape is undergoing significant transformation, particularly in developed countries where life expectancy is steadily increasing [1]. Projections indicate a substantial rise in the older adult population, especially those ages 80–89 (octogenarians). In the United States, this segment of the population is expected to more than double, escalating from 9 million in 2000 to 20 million by 2030 [2].

The impact of this aging demographic is notably reflected in healthcare utilization trends. In the years 2015–2016, the octogenarian population experienced a three-fold increase in emergency department visits compared to their younger adult counterparts [3]. This trend extends to surgical interventions, with a significant increase in the number of older adults requiring emergency surgical procedures. Predictions suggest that there will be approximately half a million additional operations in this older age group by 2030 [4]. Among these interventions, urgent or emergent laparotomies (UEL) are associated with significant morbidity, mortality, and reduced quality of life, especially within octogenarians [5]. Patients that are older than 65-years are ten times more likely to die from UEL than younger patients [6], and octogenarians with clinical peritonitis undergoing UEL have a mortality rate of over 40% [7]. This group’s vulnerability is exacerbated by factors such as frailty, diminished physiological reserve, and the presence of multiple comorbidities [5].

Despite the growing prevalence of UEL in octogenarians, there is a paucity of research focusing on the outcomes for this demographic, especially regarding predictors of mortality post-UEL. This study sought to identify key predictors of mortality in octogenarian trauma patients undergoing UEL. This information may help providers better counsel these high-risk octogenarians and their families.

## Methods

This study was deemed exempt, and a waiver of informed consent granted by our institutional review board as it utilizes a national deidentified database. We performed a retrospective analysis of the Trauma Quality Improvement Program (TQIP) database over a 5-year period from 2017 to 2021. Patients between the ages 80–89 (octogenarians) undergoing UEL within six hours of arrival were included. This time frame was chosen to focus on the most critical and acute cases that would require surgical intervention to address injuries sustained from trauma. The primary outcome was mortality. We compared two groups: survivors and those that died.

Patient demographics were collected, including age, sex, pre-existing comorbidities, and vitals on arrival such as systolic blood pressure (SBP), heart rate (HR), and respiratory rate (RR). Comorbidities included cerebrovascular accident (CVA), dementia, congestive heart failure (CHF), myocardial infarction (MI), hypertension, peripheral artery disease (PAD), chronic obstructive pulmonary disease (COPD), chronic kidney disease (CKD), cirrhosis, diabetes, psychiatric disorders, alcohol use disorder, smoking history, anticoagulant use, and functional independence status (defined by TQIP as: ‘Pre-injury functional status may be represented by the ability of the patient to complete age-appropriate activities of daily living’). The injury profile included the mechanism of trauma, injury severity score (ISS), and injuries to specific organs. Lung injuries are defined as any traumatic lung injury including pneumothorax, hemothorax, hemopneumothorax, pulmonary contusion/hemorrhage, pulmonary laceration, and lung injury NOS (not otherwise specified). Post-operative complications collected included CVA, delirium, cardiac arrest, MI, pulmonary embolism (PE), acute respiratory distress syndrome (ARDS), unplanned intubation, ventilator-associated pneumonia (VAP), acute kidney injury (AKI), catheter associated urinary tract infection (CAUTI), deep vein thrombosis (DVT), pressure ulcer, central line associated blood stream infection (CLABSI), sepsis, and unplanned return to the operating room.

For the statistical analysis, continuous variables were compared using the Mann-Whitney-U test, while categorical variables were assessed with the chi-square test. We reported continuous data as either medians with interquartile range (IQR) or means with standard deviation, and categorical data as percentages. A multivariable logistic regression model was utilized to identify independent associated risk factors for mortality. This model included variables that were chosen by coauthor consensus after review of the literature and that were deemed significant in the univariate analysis with a p-value < 0.20 [5, 8–10]. All other p-values were two-sided, with a statistical significance level of < 0.05. All analyses were performed with IBM SPSS Statistics for Windows (Version 29, IBM Corp., Armonk, NY).

## Results

### Demographics of octogenarian trauma patients undergoing UEL

Of the 701 octogenarian trauma patients who underwent UEL, 377 (53.8%) survived and 324 (46.2%) died with 42.0% of deaths occurring within the first 24-hours of presentation. Non-survivors exhibited significantly higher rates of cirrhosis (3.5% vs. 1.1%, *p* = 0.028) and a lower rate of functional independence (6.4% vs. 12.6%, *p* = 0.007) prior to traumatic injury. Additionally, lower rates of hypertension (50.3% vs. 64.2%, *p* < 0.001) and a history of smoking (1.9% vs. 7%, *p* = 0.002) were observed in the non-survivor group, as well as higher rates of hypotension (SBP < 90 mmHg) (37.6% vs. 21.9%, *p* < 0.001) on arrival. Additionally, we conducted a comparison between American College of Surgeons designated Level I and Level II/III hospitals and found no significant difference in the rate of death (*p* = 0.13) (Table 1).

**Table 1:** Demographics of octogenarian trauma patients undergoing urgent or emergent exploratory laparotomy

	Survived	Died	
Characteristic	(*n* = 377)	(*n* = 324)	*p*-value
Age, median (IQR)	83 (5)	84 (5)	0.072
Male, n (%)	200 (53.2%)	182 (56.3%)	0.403
Comorbidities, n (%)			
Cerebrovascular accident	19 (5.1%)	19 (6.1%)	0.558
Dementia	32 (8.6%)	17 (5.4%)	0.116
Congestive heart failure	37 (9.9%)	39 (12.5%)	0.279
Myocardial infarction	9 (2.4%)	4 (1.3%)	0.285
Hypertension	240 (64.2%)	147 (50.3%)	< 0.001
Peripheral artery disease	3 (0.8%)	6 (1.9%)	0.201
COPD	37 (9.9%)	25 (8%)	0.392
Chronic kidney disease	11 (2.9%)	3 (1%)	0.069
Cirrhosis	4 (1.1%)	11 (3.5%)	0.028
Diabetes	68 (18.2%)	63 (20.2%)	0.505
Psychiatric disorder	43 (11.5%)	21 (6.7%)	0.032
Alcohol use disorder	7 (1.9%)	4 (1.3%)	0.540
Smoking	26 (7%)	6 (1.9%)	0.002
Anticoagulant use	111 (29.6)	84 (26.9%)	0.438
Functionally independent	47 (12.6%)	20 (6.4%)	0.007
Vitals on arrival, n (%)			
Tachypnea (RR > 22)	100 (26.8%)	118 (39.3%)	< 0.001
SBP < 90 mmHg	82 (21.9%)	118 (37.6%)	< 0.001
Tachycardia (HR > 120)	33 (8.8%)	30 (9.6%)	0.716
Trauma verification, n (%)			0.024
Level I	192 (65.5%)	172 (71.7%)	
Level II	85 (29.0%)	65 (27.1%)	
Level III	16 (5.5%)	3 (1.3%)	

IQR = interquartile range; COPD = chronic obstructive pulmonary disease; SBP = systolic blood pressure; HR = heart rate; RR = respiratory rate

### Injury profiles for octogenarian trauma patients undergoing UEL

Compared to octogenarian survivors, non-survivors had increased rates of motor vehicle collisions (60.2% vs. 41.9%, *p* < 0.001) and more patients required concurrent thoracotomy for hemorrhage control (7.4% vs. 0.3%, *p* < 0.001). Octogenarian non-survivors also had a higher median ISS (25 vs. 14) and more often sustained fractures to the ribs (67.3% vs. 44%), spine (42.9% vs. 24.1%), and lower extremities (24.1% vs. 13%) (all *p* < 0.001). Non-survivors also had higher rates of brain (17.3% vs. 5.6%), heart (8.6% vs. 1.6%), and lung injuries (57.4% vs. 23.9%) (all *p* < 0.001). (Table 2).

**Table 2:** Injury profiles and other interventions of octogenarian trauma patients undergoing urgent or emergent exploratory laparotomy

	Survived	Died		
Characteristic	(*n* = 377)	(*n* = 324)	*p*-value	
ISS, median (IQR)	14 (13)	25 (17)	< 0.001	
Blunt mechanism, n (%)				
Motor vehicle crash	158 (41.9%)	195 (60.2%)	< 0.001	
Fall	123 (32.6%)	48 (14.8%)	< 0.001	
Pedestrian struck	12 (3.2%)	36 (11.1%)	< 0.001	
Motorcycle accident	3 (0.8%)	2 (0.6%)	0.780	
Bicycle	1 (0.3%)	4 (1.2%)	0.128	
Penetrating mechanism, n (%)				
Knife wound	46 (12.2%)	10 (3.1%)	< 0.001	
Gunshot wound	24 (6.4%)	19 (5.9%)	0.783	
Non-laparotomy hemorrhage control surgery, n (%)				
Thoracotomy	1 (0.3%)	23 (7.4%)	< 0.001	
Sternotomy	1 (0.3%)	3 (0.9%)	0.247	
Extremity	1 (0.3%)	1 (0.3%)	0.914	
Neck	0	1 (0.3%)	0.280	
Fracture, n (%)				
Rib	166 (44%)	218 (67.3%)	< 0.001	
Spine	91 (24.1%)	139 (42.9%)	< 0.001	
Upper extremity	39 (10.3%)	49 (15.1%)	0.057	
Lower extremity	49 (13%)	78 (24.1%)	< 0.001	
Injury, n (%)				
Brain	21 (5.6%)	56 (17.3%)	< 0.001	
Heart	6 (1.6%)	28 (8.6%)	< 0.001	
Lung	90 (23.9%)	186 (57.4%)	< 0.001	
Diaphragm	25 (6.6%)	40 (12.3%)	0.009	
Liver	52 (13.8%)	81 (25%)	< 0.001	
Spleen	132 (35%)	107 (33%)	0.580	
Pancreas	12 (3.2%)	19 (5.9%)	0.085	
Kidney	16 (4.2%)	24 (7.4%)	0.072	
Bladder	10 (2.7%)	14 (4.3%)	0.226	
Stomach	13 (3.4%)	13 (4%)	0.694	
Small intestine	81 (21.5%)	78 (24.1%)	0.415	
Colon	65 (17.2%)	48 (14.8%)	0.384	
Rectum	4 (1.1%)	1 (0.3%)	0.238	

ISS = injury severity score; IQR = interquartile

### In-hospital complications for octogenarian trauma patients undergoing UEL

Non-survivors experienced increased overall complications (46.0% vs. 22.3%, *p* < 0.001) and specifically AKI (8.0% vs. 1.9%), cardiac arrest (27.8% vs. 1.9%), ARDS (1.2% vs. 0%, *p* = 0.03), sepsis (3.1% vs. 0.3%, *p* = 0.003), and unplanned return to the operating room (4.4% vs. 1.6%, *p* = 0.028) (Table 3).

**Table 3:** Complications of octogenarian trauma patients undergoing urgent or emergent exploratory laparotomy

	Survived		Died		
Characteristic	(*n* = 377)		(*n* = 324)		*p*-value
Complications, n (%)	84 (22.3%)		149 (46%)		< 0.001
Cerebrovascular accident	3 (0.8%)		7 (2.2%)		0.129
Delirium	8 (9.2%)		1 (1.5%)		0.048
Cardiac arrest	7 (1.9%)		90 (27.8%)		< 0.001
Myocardial infarction	5 (1.3%)		11 (3.4%)		0.067
Pulmonary embolism	4 (1.1%)		2 (0.6%)		0.525
ARDS	0		4 (1.2%)		0.030
Unplanned intubation		22 (5.8%)		22 (6.8%)	0.603
VAP	4 (1.1%)		3 (0.9%)		0.858
Acute kidney injury	7 (1.9%)		26 (8%)		< 0.001
CAUTI	5 (1.3%)		2 (0.6%)		0.347
Deep vein thrombosis	15 (4%)		4 (1.2%)		0.026
CLABSI	2 (0.5%)		2 (0.6%)		0.879
Sepsis	1 (0.3%)		10 (3.1%)		0.003
Unplanned return to OR	6 (1.6%)		14 (4.4%)		0.028
Death timing, n (%)					
< 24-hours	-		136 (42.0%)		-
24-48-hours	-		60 (18.5%)		-
>48-hours	-		128 (39.5%)		-

ARDS = acute respiratory distress syndrome; CAUTI = catheter associated urinary tract infection; CLABSI = central line associated blood stream infection; OR = operating room; VAP = ventilator-associated pneumonia

### Analysis for risk of mortality in octogenarian trauma patients undergoing UEL

On multivariable analysis the strongest independent associated risk factors for mortality in octogenarians undergoing UEL were cirrhosis (OR 8.28, CI 2.25–30.46, *p* = 0.001), lung injury (OR 2.88, CI 1.87–4.42, *p* < 0.001), brain injury (OR 2.23, CI 1.20–4.13, *p* = 0.01), and hypotension on arrival (OR 1.83, CI 1.22–2.75, *p* = 0.004). Functional independence status and mechanism of trauma were not independently associated with mortality (all *p* > 0.05) (Table 4).

**Table 4:** Multivariable logistical regression analysis for risk of mortality in octogenarian trauma patients undergoing urgent or emergent exploratory laparotomy

		95% CI for OR		
Variable	OR	Lower	Upper	*p*-value
Comorbidities				
Chronic kidney disease	0.479	0.12	1.96	0.306
Cirrhosis	8.591	2.35	31.39	0.001
Hypertension	0.741	0.51	1.01	0.115
Smoking	0.208	0.06	0.68	0.009
Functionally independent	0.565	0.29	1.10	0.094
Vitals on arrival				
SBP < 90mmHg	1.794	1.19	2.71	0.005
Tachypnea (RR > 22)	1.789	1.21	2.64	0.003
Tachycardia (HR > 120)	0.307	0.72	0.38	1.352
Blunt mechanism	1.450	0.76	2.77	0.261
Thoracotomy	16.586	2.07	132.76	0.008
Injury				
Brain	2.271	1.22	4.24	0.010
Heart	2.667	0.97	7.31	0.056
Lung	2.781	1.80	4.30	< 0.001
Diaphragm	1.550	0.82	2.94	0.179
Spleen	0.786	0.52	2.95	0.245
Pancreas	2.477	1.04	5.90	0.041
Kidney	1.304	0.58	2.95	0.524
Fracture				
Upper extremity	0.618	0.34	1.12	0.111
Lower extremity	1.159	0.70	1.93	0.570

OR = odds ratio; CI = confidence interval; SBP = systolic blood pressure; HR = heart rate; RR = respiratory rate

## Discussion

As the global population continues to age, the proportion of octogenarians sustaining trauma is increasing. This national analysis highlights the complexities of managing octogenarian trauma patients, revealing that nearly half died. Furthermore, a comprehensive multivariable logistic regression analysis identified independent associated risk factors for death including comorbidities (e.g., cirrhosis) and specific injuries (i.e., brain and lung injuries). Vitals on arrival were found to be associated risk factors for death, highlighting the increased risk when this patient population arrives in shock. Concurrent thoracotomy for hemorrhage control increased risk of death by over 16-fold. Interestingly, other factors like functional independence were not associated with mortality independently. This suggests that underlying health conditions, rather than functional status alone, play a critical role in determining outcomes in this vulnerable population.

The liver has a vital role in the physiologic response to traumatic injury, performing protein synthesis, detoxification, and immunologic response. Cirrhosis is a progressive liver disease and brings with it a myriad of metabolic and nutritional imbalances, profoundly altering the body’s response to trauma, including coagulopathy. Cirrhosis has been identified as an independent risk factor for mortality in various trauma populations [11–17]. Cirrhotic patients have a six-fold higher risk of mortality after traumatic lung injury [17], a two-fold increased risk of mortality in the setting of traumatic brain injury [18], and a six-fold risk of death following trauma [19], compared to non-cirrhotic patients. This current study of octogenarians found cirrhosis to be the strongest independent associated risk factor for death, with an over eight-fold increase compared to non-cirrhotic octogenarians undergoing UEL. Trauma patients have a high rate of cirrhosis comparative to other populations due to the known link between alcohol consumption and traumatic injury [20]. While the necessity for an UEL in trauma cases is often dictated by life-threatening conditions like severe bleeding, the presence of cirrhosis should be a significant consideration in the counseling of prognostication and overall management strategy. This is especially crucial in the postoperative and recovery phases, where cirrhosis management can greatly influence patient outcomes [21]. Future research is needed to ascertain if risk can be mitigated by involvement of multidisciplinary teams and/or at liver centers of excellence, which have been demonstrated to improve other outcomes related to patients with cirrhosis [22].

While comorbidities can affect all age groups, functional independence is a preoperative factor that is receiving increasing attention in older adult populations such as octogenarians as it is highly related to frailty. In addition, functional outcomes after trauma have been widely studied to predict long-term mortality [23–25]. Similarly, there are recent studies that found frailty as a predictor of mortality and morbidity in older trauma patients [26–28]. The definition of frailty, while widely varied, is a clinical condition that characterizes the overall decreased physiologic reserve and increased vulnerability [26]. There are some studies that utilized frailty measures that specifically focused on activities of daily living and functional status that found an increased association with post-operative mortality and complications [26–28]. Despite these findings, this current study revealed that while octogenarians who survived UEL generally had better functional status, functional independence prior to trauma was not independently associated with mortality after controlling for various confounders such as comorbidities and specific injuries (i.e., brain and lung injuries). This suggests that while assessing functional status prior to UEL is valuable, it should not be the sole determinant in deciding the course of treatment for octogenarians suffering trauma requiring UEL. Instead, a more holistic approach in evaluating these patients is needed and should incorporate the various risk factors identified in this study.

Specific injury patterns can also impact mortality risk after surgical intervention for trauma patients. This study aligns with multiple studies that demonstrates a strong correlation between head injuries and increased risk of mortality and morbidity in older populations [29–31]. However, our study also demonstrated that intra-thoracic injuries significantly impacted mortality, particularly when necessitating a thoracotomy for hemorrhage control. The need for thoracotomy typically indicates a high-energy mechanism of injury and severe trauma, which are associated with extensive blood loss and the need for rapid, complex surgical intervention. The combination of these factors results in a markedly higher mortality risk. Interestingly, we found that lung injury had the highest association with mortality, even surpassing the impact of injuries to the head or heart. This heightened risk can be attributed to several factors unique to lung injuries and the physiology of octogenarians. Firstly, lung injuries, which include a range of conditions such as pneumothorax, hemothorax, and pulmonary contusion, often lead to compromised respiratory function. Pre-existing diminished lung capacity and reduced ventilatory response to hypoxia exacerbate this issue, making octogenarians more susceptible to respiratory failure post-trauma. Secondly, lung injuries in older adults are linked to longer hospital and ICU stays, and increased ventilator dependence. These complications can be particularly challenging to manage in octogenarians, who generally have less physiological reserve compared to younger patients. Therefore, the combination of the intrinsic severity of lung injuries and the physiological vulnerability of octogenarians explains why lung injuries pose such a significant risk of mortality in this age group.

Limitations of this study include those inherent to large database studies, such as selection and reporting bias. Additionally, the TQIP database does not account for variations in initial management approaches, which can vary significantly based on individual physician or hospital protocols. This heterogeneity in treatment can influence patient outcomes but is not captured in the database. Furthermore, the database does not employ an objective or even standardized functional independence scale or calculation. Instead, it relies on chart reviews to determine if a patient was functionally independent or not based on documentation, which introduces a potential for inconsistency in the assessment of functional status. Other limitations to consider include the lack of detailed information on frailty (e.g., Trauma Specific Frailty Index) and postoperative care and rehabilitation, which are crucial aspects of recovery, especially for elderly patients. The database also does not provide information on long-term outcomes or patient centric outcomes, such as quality of life, long-term morbidity, or functional status post-discharge, which are important for a comprehensive understanding of the impact of UEL in octogenarians. Moreover, the retrospective study design inherently limits the ability to establish causality between identified associated risk factors and patient outcomes.

## Conclusions

In this large national analysis spanning five years of data, cirrhosis was the strongest independent associated risk factor for mortality in octogenarians suffering traumatic injury undergoing UEL. Lung injuries were found to be a stronger independent associated risk factor for mortality than head injuries. After controlling for confounders, this study did not identify pre-trauma functional status as an independent predictor of mortality in this population. These findings can help inform discussions about prognostication for octogenarians undergoing UEL.

## Data Availability

No datasets were generated or analysed during the current study.
